# cvlr: finding heterogeneously methylated genomic regions using ONT reads

**DOI:** 10.1093/bioadv/vbac101

**Published:** 2023-01-23

**Authors:** Emanuele Raineri, Mariona Alberola i Pla, Marc Dabad, Simon Heath

**Affiliations:** CNAG-CRG, Centre for Genomic Regulation (CRG), Barcelona Institute of Science and Technology (BIST), Barcelona 08028, Spain; CNAG-CRG, Centre for Genomic Regulation (CRG), Barcelona Institute of Science and Technology (BIST), Barcelona 08028, Spain; CNAG-CRG, Centre for Genomic Regulation (CRG), Barcelona Institute of Science and Technology (BIST), Barcelona 08028, Spain; CNAG-CRG, Centre for Genomic Regulation (CRG), Barcelona Institute of Science and Technology (BIST), Barcelona 08028, Spain

## Abstract

**Summary:**

Nanopore reads encode information on the methylation status of cytosines in CpG dinucleotides. The length of the reads makes it comparatively easy to look at patterns consisting of multiple loci; here, we exploit this property to search for regions where one can define subpopulations of molecules based on methylation patterns. As an example, we run our clustering algorithm on known imprinted genes; we also scan chromosome 15 looking for windows corresponding to heterogeneous methylation. Our software can also compute the covariance of methylation across these regions while keeping into account the mixture of different types of reads.

**Availability and implementation:**

https://github.com/EmanueleRaineri/cvlr.

**Contact:**

simon.heath@cnag.crg.eu

**Supplementary information:**

Supplementary data are available at *Bioinformatics Advances* online.

## 1 Introduction

In this article, we exploit the fact that one can measure both DNA sequence and cytosine methylation status in one go via Nanopore sequencing (Simpson *et al.*, 2017) to scan the genome looking for regions where methylation changes between DNA molecules. The idea of quantifying the variability in binary epigenetic patterns in single reads was initially discussed in Landan *et al.* (2012). A probabilistic model to study allele-specific methylation has been proposed by Fang *et al.* (2012) on short reads measured with whole genome bisulfite sequencing; Barrett *et al.* (2017) discuss the analysis of RRBS libraries and Abante *et al.* (2020) present a statistical method for finding haplotype-specific methylation through long reads based on an Ising-like model but as far as we know there is no method for the analysis of methylation heterogeneity on long reads which does not rely on phasing, which is also used in Akbari *et al.* (2021) from where we take some benchmarking data. What we propose here is a software which can be run from the command line on the output of (among others) Nanopore sequencing experiments to cluster reads based on methylation patterns appearing on them. Since it does not rely on a previous phasing step, our method can capture allele-specific differences but also other forms of heterogeneity, e.g. due to cell subpopulations. We show some results obtained by looking at chromosome 15 (chr15), which we chose because it contains a number of well-studied imprinted regions Reik and Walter (2001). Our test dataset is the sample NA12878 (Lymphoblastoid cell line) sequenced by the Nanopore consortium (Jain *et al.*, 2018).

## 2 Results and methods

### 2.1 Known imprinted genes

**Fig. 1.**
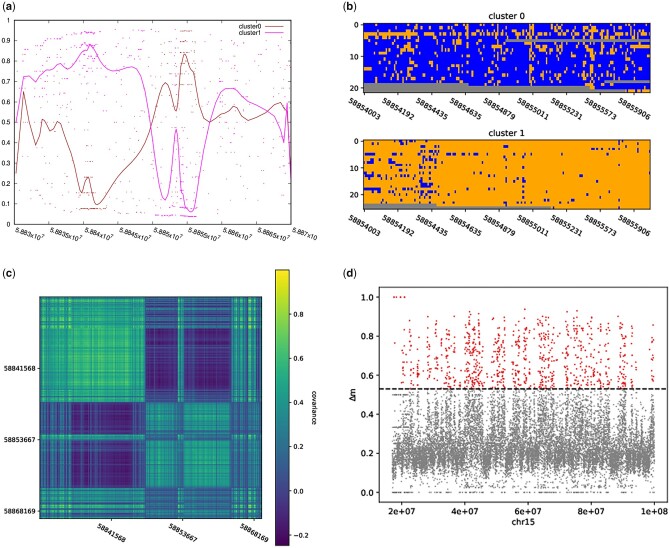
(**a**) Mean values of methylation in the two clusters over the GNAS region. Single dots represent methylation levels (colored by cluster), the continuous lines are obtained by smoothing with Bezier curves. (**b**) The two corresponding subpopulations of reads in the subregion 58854000–58856000 (blue = methylated, orange = not methylated, gray = not known). (**c**) Covariance matrix of the methylation in the GNAS gene (see explanation in Section 2.3). (**d**) Scan of chr15 in windows of 5* *kb. For each window, we computed the clustering (*k *=* *2) of the reads and the median absolute difference in methylation across clusters, and plotted it along the chromosome. In the main text, we define as peaks the values above the horizontal line at ≈0.53

To give an example on how to look at allele-specific methylation, we first considered some genes known to have imprinted expression in many tissues (Table 1). An example of analysis on GNAS is in Figure 1. Supplementary material contains more examples. For each gene, we clustered in *k *=* *2 groups the reads mapping on the corresponding genomic stretch and also tagged each read with its haplotype as computed by WhatsHap (Martin *et al.*, 2016). Haplotypes for each read are computed by phasing the entire chromosome containing the considered locus. We then check that each cluster is enriched in a specific haplotype. In some cases, we do not look at the entire gene region, rather we select stretches where the methylation seems to be at intermediate levels or positions surrounding known regulatory sites, or already described as imprinting control regions in Joshi *et al.* (2016) and Court *et al.* (2014) as reported in Akbari *et al.* (2021); the exact loci are under the ‘chr:start-end’ heading and known imprinted regions have a ‘dmr’ in their name.

**Table 1.** Known imprinted genes we looked at

GENE	chr:start–end	Δm	*P*val (Δm)	*P*val (phasing)
GNAS	chr20:58830000–58870000	0.667	$<10^{-3}$	8.2e−30
H19dmr	chr11:1997582–2003510	0.609	$<10^{-3}$	0.0079
H19	chr11:1995163–2001470	0.474	$<10^{-3}$	1
PEG10	chr7:94656325–94669695	0.457	$<10^{-3}$	3.2e−08
MEG3	chr14:100820000–100840000	0.231	$<10^{-3}$	3.8e−07
SNRPN	chr15:24950000–24958000	0.637	$<10^{-3}$	1.4e−10
IGF2	chr11:2132761–2133882	0.200	$<10^{-3}$	0.0031
PLAGL1	chr6:144000000–144020000	0.574	$<10^{-3}$	2.7e−07

*Note*: For each region, we repeat the clustering with different seeds for the random initialization and we pick the output with the best likelihood. The third column reports the median of the absolute difference in methylation between clusters; we compute its empirical *P*-value (fourth column) using its distribution on a set of 1000 random cluster assignments. We also check that each cluster is enriched in a specific haplotype via a Fisher’s test (*P*-value in the fifth column).

### 2.2 Scan of chr15

We then scanned chr15 in windows of 5 kb and clustered (with *k *=* *2) the reads mapping on each window; the median absolute difference in methylation between the clusters at each locus is in Figure 1d. The 95% percentile of the distribution of median absolute difference in methylation between cluster is 0.53 which we use a threshold for further analysis below. To compare the results of the clustering with the output of WhatsHap, we haplotagged all the reads mapping onto chr15 and computed the median absolute methylation difference between haplotypes in windows of 5 kb. There were 17 windows where cvlr could not give results and WhatsHap could, and 905 windows where WhatsHap did not give any result and cvlr did. The threshold of 0.53 gives 909 peaks when using cvlr and ≈40 windows when using WhatsHap (see the Supplementary material for further details). When running Whatshap to haplotag reads over chr15, we find that 100 920 reads are assigned to haplotype H1, 97 470 to haplotype H2 and for 130 963 reads (around 40% of the total), it is not possible to determine the haplotype they belong to probably due to the sparsity of heterozygous SNPs. Using the same set of 5k windows, we checked the correspondence between phasing and methylation; to this end, we computed the *P*-value of a Fisher’s test on all windows so that positions where a certain haplotype is enriched in a certain cluster correspond to a low *P*-value. Supplementary Figure S2 shows the negative logarithm of the Fisher *P*-value at each genomic window; we also highlight the positions of imprinted control regions described in Joshi *et al.* (2016) and Court *et al.* (2014) which all (but one) fall into peaks, We compared the coordinates of 909 peaks with the coordinates of NA12878 enhancers, and we obtain 55 intersections; this corresponds (by randomization) to a *P*-value of <1%. We intersect those peaks with genes to find 472 overlapping genes which is not significant at the 5% level.

### 2.3 Methods

The software we used to produce Figure 1 is called cvlr, and it consists of three executables, cvlr-cluster which runs the clustering algorithm, cvlr-meth-of-bam which helps in extracting methylation information from BAM files and cvlr-stats which processes the clusters to compute descriptive statistics. It is not necessary to use cvlr-meth-of-bam to produce the input for cvlr-cluster, users can equally adapt the output of e.g. Nanopolish Simpson. Internally, the algorithm sees the data as a binary matrix (with missing data) *X*, with *n* rows representing reads and *d* columns corresponding to genomic positions. Each position *X_ij_* can be 0 (unmethylated) 1 (methylated) or −1 (unknown). Reads are clustered (into *k* clusters) via a mixture of multivariate Bernoulli distributions. To this purpose, we use an Expectation–Maximization (EM) algorithm (Dempster *et al.*, 1977) allowing for incomplete observations; the output of the algorithm is:

- a vector *π_l_* which contains the weight of each component of the mixture for l∈1,…,k. One has $\sum_{l=1}^{k}\pi_{l}=1$.
- a *k* × *d* matrix *μ* containing the average methylation for each cluster at each position. We indicate the *l*th row of *μ* with *μ_l_* and one has $E[x]=\sum_{l=1}^{k}\pi_{l}\mu_{l}$, where E[x] is a vector of dimension *d*.
- the *d* × *d* covariance matrix of the methylation computed as
  $$\sum_{l=1}^{k}\pi_{l}\{\Sigma_{l}+\mu_{l}\mu_{l}^{T}\}-E[x]E{\left[x\right]}^{T},$$

where $\Sigma_{l}=\text{diag}(\mu_{l}(1-\mu_{l}))$ see Bishop and Nasrabadi (2006).

The E step computes the posterior probability that a given read belongs to a certain cluster; the M step updates the *μ* matrix, replacing missing data with a weighted sum of the corresponding positions in the old *μ*. cvlr-stats computes, among other things, the median absolute difference in methylation and the posterior probability of difference in methylation between all pairs of clusters across all the positions (in pairs) using the method described in Raineri *et al.* (2014). The command line allows to specify the number of clusters, a seed for the random initialization of the algorithm and the maximum number of iterations of the EM algorithm.

To verify that the software works as expected, we simulate 100 reads covering 1000 positions; methylation values are generated first by assuming that the reads form two clusters corresponding to an uniformly low and uniformly high methylation levels. We then check that the simulated clusters are recovered correctly by cvlr-cluster, even in the presence of missing values. We repeat the same procedure with clusters formed by reads which are methylated or not methylated at alternating (non-overlapping) positions. The error rate is ≈2% in all these tests.

We downloaded all the test data we used from wgs and processed them with our internal pipeline which is based on Megalodon meg. Enhancers were taken from Gao and Qian (2020). In summary, we showed how to use cvlr to measure methylation heterogeneity along the human genome; although we used imprinted regions (and hence allele-specific methylation) as a benchmark, cvlr can be run to detect subpopulation of reads regardless of whether they are due to an allelic effect and does not need a preliminary phasing step. As mentioned above, this is important in practice because when phasing many reads cannot be assigned to an haplotype simply because they contain no informative SNPs; on the other hand, methylation measurements are dense along the genome and each read bears a methylation pattern.

## Supplementary Material

vbac101_Supplementary_Data

## Data Availability

Data derived from a source in the public domain which is referenced in the paper.
