# Supplementary material for: cvlr: finding heterogeneously methylated genomic regions using ONT reads
Source: Bioinform Adv. 2023 Jan 23;3(1):vbac101. doi: 10.1093/bioadv/vbac101 (PMC9887406; doi:10.1093/bioadv/vbac101)
Supplement: vbac101_Supplementary_Data [file vbac101_supplementary_data.pdf]

1 cvlr : usage

The user needs to have a BAM file containing methylation data encoded with the Mm/Ml tags as explained in the SAM specifications. This is also the standard output format (at the moment) of Nanopore's Megalodon. First one can create the methylation matrix from the BAM file with (for example)

```
cvlr-meth-of-bam NA12878.cram chr20:58839718-5891119 >
GNAS-matrix.txt
```

then one clusters the reads with

```
cvlr-cluster GNAS-matrix.txt 2 1 100 > GNAS-clusters.txt
```

(This creates 2 clusters, with seed 1 for the random numbers generator and a maximum of 100 EM iterations). There is a post-processing script (cvlr-stats.py) which can be used to look at the clusters (for example for plotting the methylation values)

```
cvlr-stats.py GNAS-clusters.txt GNAS-matrix.txt >
GNAS-stats.txt
```

In the output file of cvlr-stats columns $1, 4, 7$ contain respectively the genomic position, methylation in cluster 0 and methylation in cluster 1. cvlr-stats has different options, including for plotting the covariance matrix

```
cvlr-stats.py --outputfn GNAS-clusters-cov.png
GNAS-clusters.txt
```

1.1 Timings

Over 10 measurements cvlr (including extracting methylation from bam files and clustering) takes an average of $1.101s(\sigma : 0.006s)$ to analyze the region chr20:58830000-58870000 (GNAS); For reference, Whatshap uses $1.263s(\sigma : 0.012s)$ to haplotag the same region. In a more realistic set up where the output of cvlr is passed to the analysis script cvlr-stats.py (eg to prepare tables for plotting) the cvlr pipeline uses $1.992s(\sigma : 0.029s)$ to complete the computation.

1.2 Example of megalodon usage

To run cvlr one needs to first extract long read methylation information. Here below we show the Megalodon command line we used to work with FAST5 files generated by ONT sequencing.

```
megalodon <flowcell> \
      --guppy-params "-d <model directory>"  \
      --guppy-config res_dna_r941_min_modbases_5mC_CpG_v001.cfg \
      --outputs basecalls mappings mod_mappings mods \
      --reference <hg38 reference fasta> \
      --mod-motif m CG 0 \
      --devices 0 \
      --processes 5 --guppy-timeout 300 \
      --guppy-server-path <path to guppy> \
      --output-directory <output-dir> \
      --overwrite 2> <log file per flowcell>
```

2 Effects of random initialization on clustering

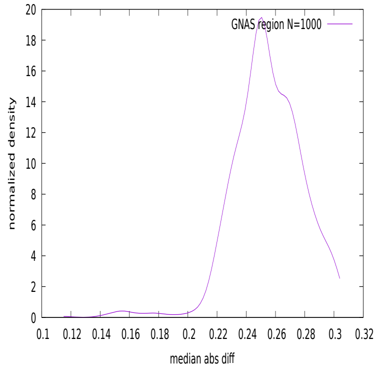

Figure 1: Effect of different random initializations on the median absolute difference in methylation between $k = 2$ clusters in the GNAS region. Mean 0.25, standard deviation 0.02.

3 Binomial p-value on the sizes of the clusters

When looking at putatively imprinted regions, we test that the two clusters have approximately equal size. To this purpose, let m be the size of the biggest cluster; the null hypothesys is that the clusters have equal equal size and hence the probability that one obtains a size m or bigger is given by: $\sum_{s \geq m} \binom{N}{s} (1/2)^N$. For the genes listed in the main manuscript we obtain the p-values listed in supplementary table 1.

GENE	cluster0	cluster1	pval
GNAS	95	76	0.084
H19dmr	21	17	0.314
H19	5	29	0.000
PEG10	68	26	0.000
MEG3	46	66	0.036
SNRPNdmr	24	34	0.119
IGF2dmr2	19	10	0.068
PLAGL1	64	45	0.042

Table 1

4 Fisher test against haplotagging

We test the enrichement for each cluster in a specif haplotype using Fisher's test.

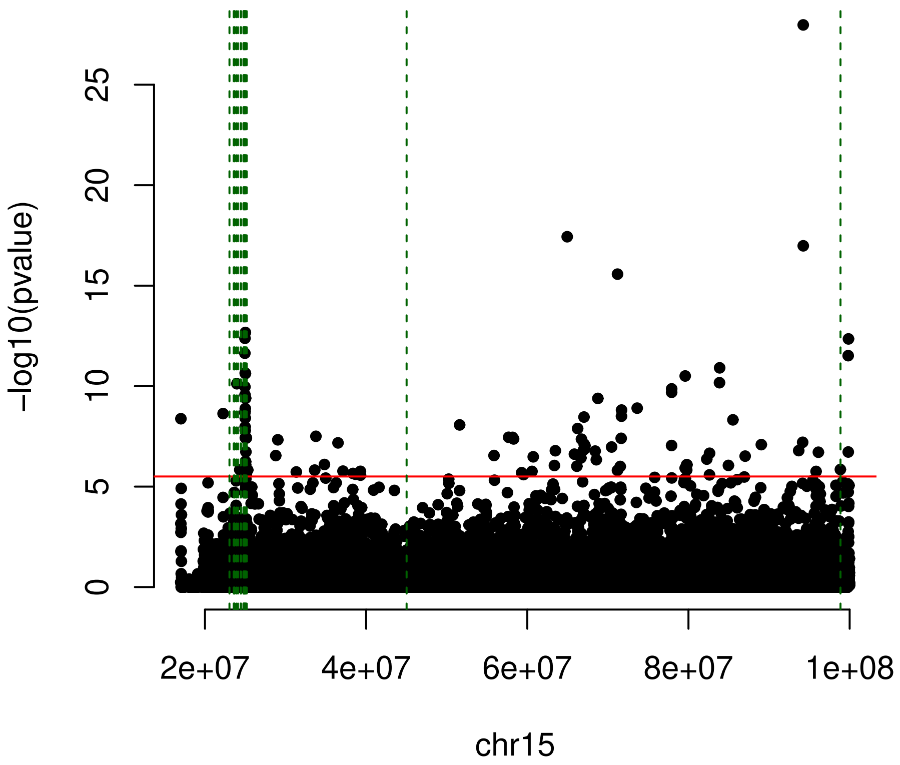

Figure 2: Negative logarithm of the Fisher p-value at each genomic window; we also highlight the positions of imprinted control regions described in [1].

5 Median absolute difference

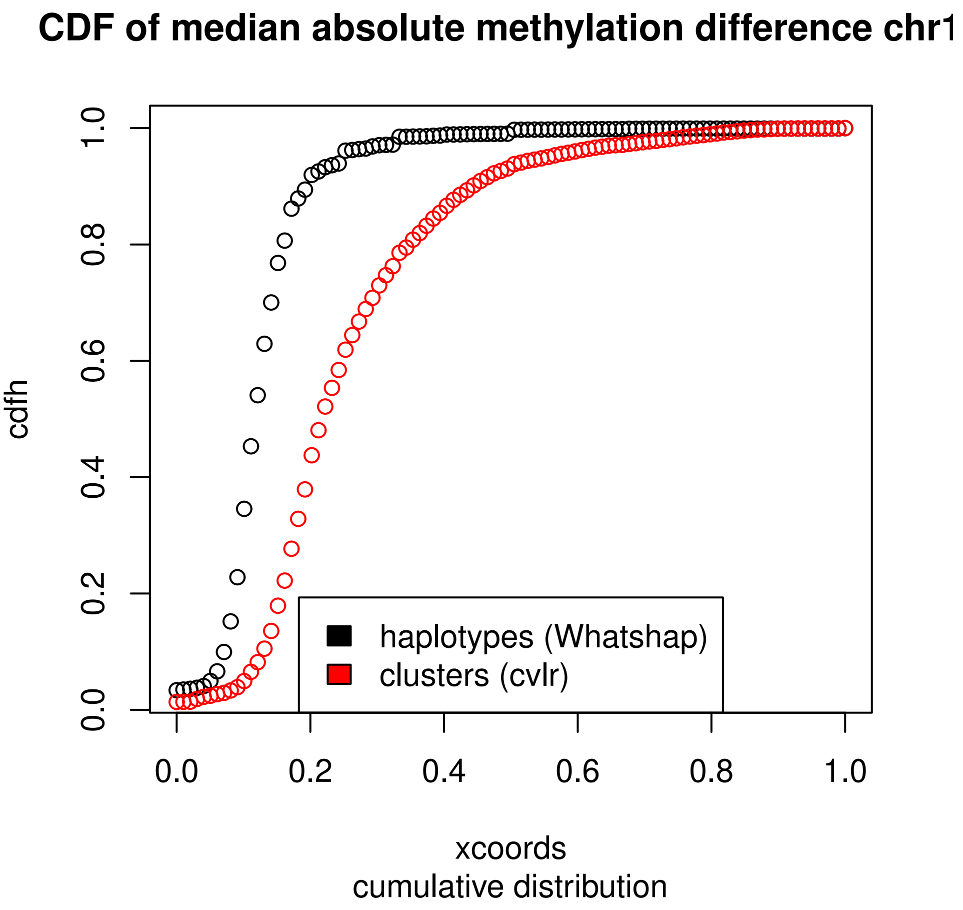

Figure 3: Distribution of median absolute difference in methylation when clustering according to haplotag or according to methylation patterns.

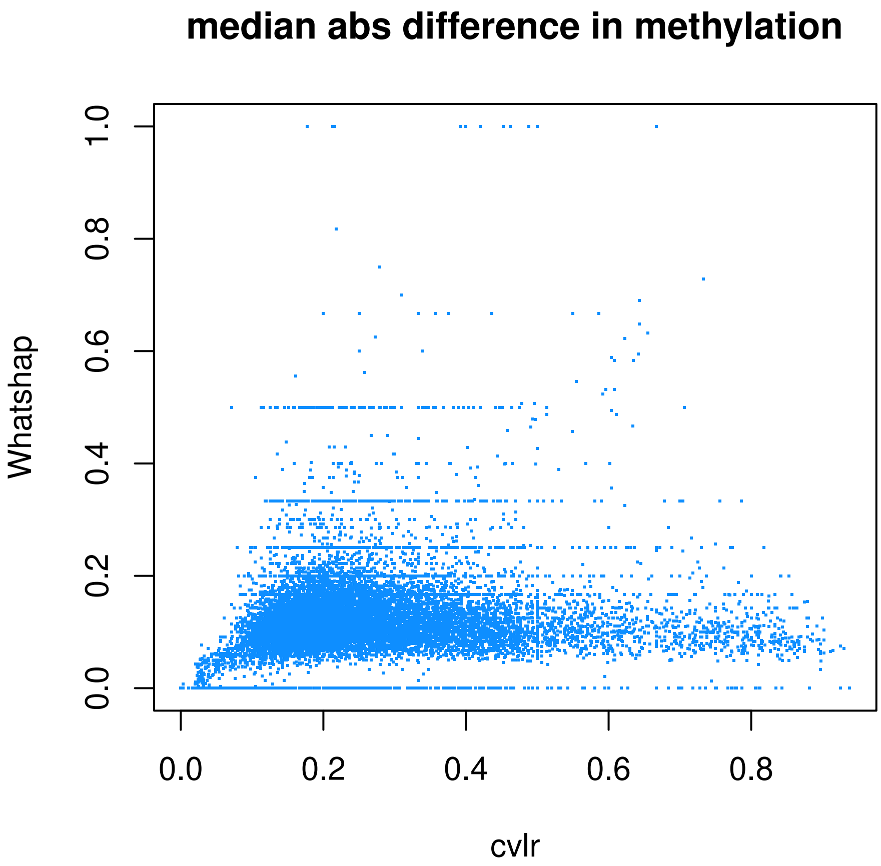

Figure 4: Median absolute difference in methylation, window by window, WhatsHap vs cvlr.

6 Mixture of multivariate bernoulli distribution

A mixture of multivariate Bernoulli distributions has the form

$$P(x_{ij}|\mu_{kj},\pi_k) = \prod_{i=1}^{N}\sum_{l=1}^{K}\pi_k\prod_{j=1}^{D}\mu_{kj}^{x_{ij}}(1-\mu_{kj})^{(1-x_{ij})}$$

where $\sum_k \pi_k = 1$ and $x_{ij} \in \{0,1\}$.

6.1 likelihood

$$P(x|\theta) = \prod_{i=1}^{N} p(x_i|\mu) = \prod_{i=1}^{N}\sum_{l=1}^{K}\pi_l p(x_i|\mu_l) \tag{1}$$

Now, x_i is a vector of dimension D, hence the above is equal to

$$\prod_{i=1}^{N}\sum_{l=1}^{K}\pi_l\prod_{j=1}^{D}p(x_{ij}|\mu_{lj})$$

Taking the logarithm

$$\log P(x|\theta) = \sum_{i=1}^{N}\log\left(\sum_{l=1}^{K}\overbrace{\pi_l\prod_{j=1}^{D}p(x_{ij}|\mu_{lj})}^{\phi}\right) \tag{2}$$

Where we introduce

$$\phi(\pi_l, x_i, \mu_l) = \pi_l\prod_{j=1}^{D}p(x_{ij}|\mu_{lj})$$

Hence we can rewrite (2) as

$$\sum_{i=1}^{N}\log\left(\sum_{l=1}^{K}\phi(\pi_l, x_i, \mu_l)\right)$$

which is equal to

$$\sum_{i=1}^{N}\log\left(\sum_{l=1}^{K}\exp\log\phi(\pi_l, x_i, \mu_l)\right) \tag{3}$$

If we introduce the `logsumexp` function

$$\mathrm{lse}(s_1,\ldots,s_k) = \log(\exp s_1 + \ldots + \exp s_k) = \log(\sum_k \exp s_k)$$

we can rewrite (3) as

$$\sum_{i=1}^{N} \mathrm{lse}(\log\phi(\pi_1, x_i, \mu_1), \ldots, \log\phi(\pi_K, x_i, \mu_K)) \tag{4}$$

7 Clusters for genes in table 1

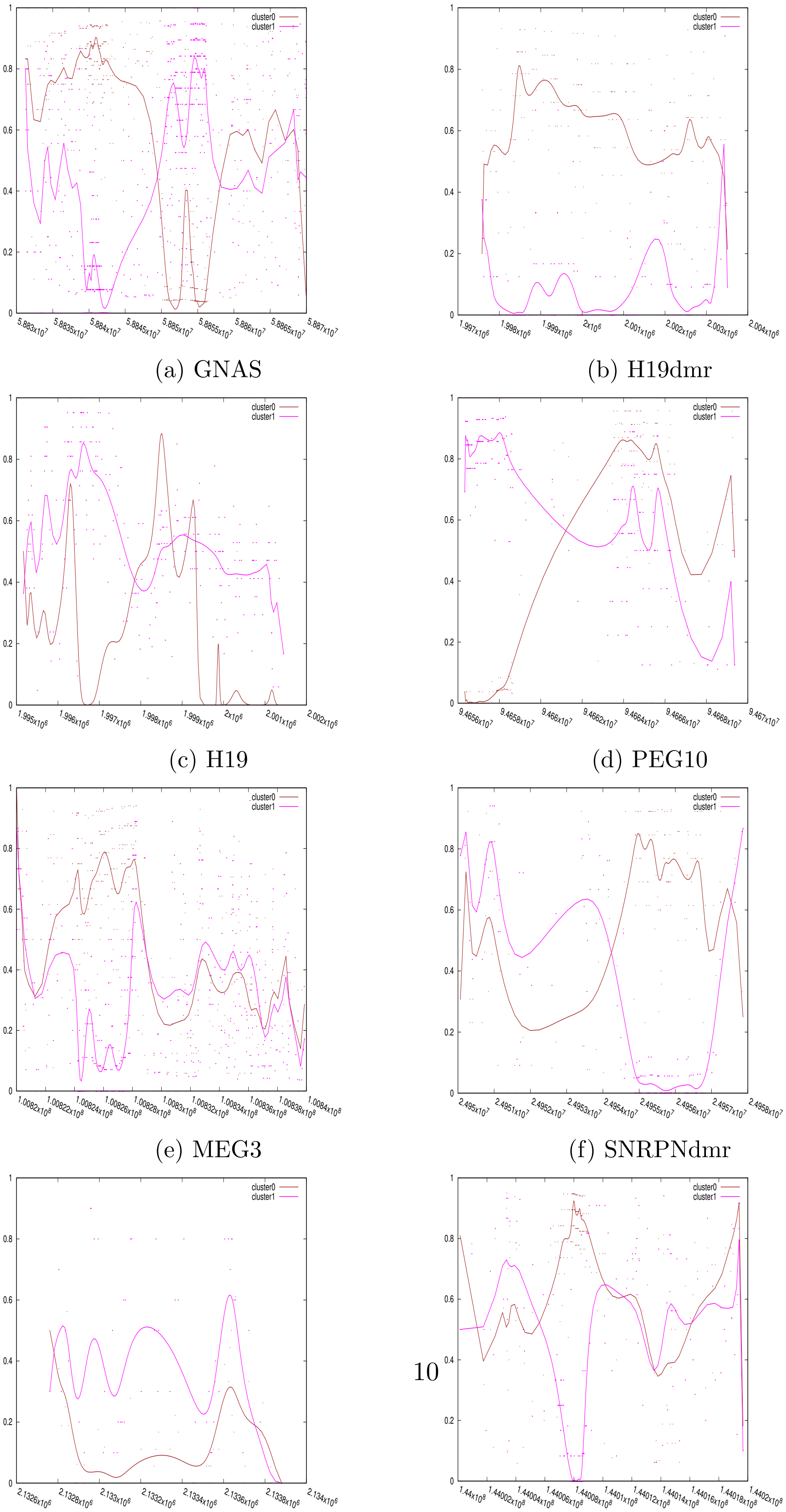

(a) GNAS (b) H19dmr

(c) H19 (d) PEG10

(e) MEG3 (f) SNRPNdmr

(g) IGF2dmr2 (h) PLAGL1

Figure 5: all the genes

References

[1] Vahid Akbari, Jean-Michel Garant, Kieran O'Neill, Pawan Pandoh, Richard Moore, Marco A Marra, Martin Hirst, and Steven JM Jones. Megabase-scale methylation phasing using nanopore long reads and nanomethphase. *Genome biology*, 22(1):1–21, 2021.
